# Detection of rabies antibodies in wild boars in north-east Romania by a rabies ELISA test

**DOI:** 10.1186/s12917-019-2209-x

**Published:** 2019-12-21

**Authors:** Mihaela Anca Dascalu, Marine Wasniewski, Evelyne Picard-Meyer, Alexandre Servat, Florentina Daraban Bocaneti, Oana Irina Tanase, Elena Velescu, Florence Cliquet

**Affiliations:** 1 Department of Public Health, Faculty of Veterinary Medicine, “Ion Ionescu de la Brad” University of Agricultural Sciences and Veterinary Medicine, Mihail Sadoveanu Alley, No. 8, 700489 Iasi, Romania; 2 ANSES, Nancy Laboratory for Rabies and Wildlife, WHO Collaborating Centre for Research and Management in Zoonoses Control, OIE Reference Laboratory for Rabies, European Union Reference Laboratory for Rabies, European Union Reference Laboratory for Rabies Serology, Technopôle Agricole et Vétérinaire, CS 40009, 54220 Malzéville, France

**Keywords:** Rabies antibody, ELISA, FAVN test, Oral vaccination, Romania, Wild boar

## Abstract

**Background:**

In the last few decades, Romania has been considered one of the European countries most affected by animal rabies, but a combination of oral rabies vaccination (ORV) campaigns in foxes alongside mandatory vaccination of pets has substantially decreased the number of rabies cases in recent years.

The objective of this study was to detect rabies antibodies in wild boar serum and thoracic fluid samples collected during the hunting season after ORV campaigns in north-eastern Romania in order to identify if wild boars are substantial competitors to foxes for ORV baits.

**Results:**

When the 312 wild boar samples were tested by ELISA (BioPro ELISA, Czech Republic), 42.31% (132/312) demonstrated rabies antibodies. In order to compare these wild boar results in terms of the percentage of immunisation, fox samples were also included in the study, and in this case only 28.40% (98/345) demonstrated rabies antibodies by ELISA. To check the diagnostic sensitivity and specificity of this ELISA, those samples with a sufficient volume from both species that had tested either negative or positive with an initial ELISA were then tested with the Fluorescent Antibody Virus Neutralisation (FAVN) assay. The overall concordance between the BioPro ELISA and FAVN test was 74.26% (75/101) in wild boar samples and 65.66% (65/99) in fox samples, 140 out of 200 samples being correlated with the two methods, although no significant statistical difference (*p* = 0.218) between the two species was registered. We found a good agreement by both tests for the ELISA-positive samples (91.30%), however the situation was different for the ELISA-negative samples, where a low agreement was demonstrated (41.18%).

**Conclusions:**

This study reports for the first time the presence of rabies antibodies in wild boar samples collected during the hunting season in Romania after ORV campaigns in rabies endemic areas. It is also the first study to demonstrate that ELISA BioPro can be used on wild boar samples with satisfactory results compared to the FAVN test for this species.

## Background

Rabies, a lethal zoonotic disease known for centuries, is caused by a virus belonging to the order *Mononegavirales*, family *Rhabdoviridae*, genus *Lyssavirus* [1], which affects warm blooded mammals and humans [2]. All around the world, species from both the *Carnivora* and *Chiroptera* orders are reservoir hosts of different variants of the rabies virus [3].

In Europe, the main rabies reservoir is represented by the red fox (*Vulpes vulpes*) [4], followed by the raccoon dog (*Nyctereutes procyonoides*) [5].

Over time, attempts to decimate the fox population in some European countries have not succeeded in reducing the incidence of rabies [6]. The only effective method in controlling the disease in Europe consists of oral rabies vaccination (ORV) through the distribution of vaccine baits in the habitat of these two species [7, 8].

The development of safe, effective vaccines incorporated into attractive baits led to the first oral vaccination programme targeting foxes. This programme was implemented in 1978 in Switzerland [7]. In 1996, after considerable efforts, Switzerland became the first rabies-free country in Europe [9–11]. The Swiss example was followed in 2001 by Belgium, France and Luxembourg [8, 12], then other central, western and northern European countries [13–19].

Following these good results, numerous eastern and southern European countries have launched ORV programmes, which have been followed by a significant decrease in positive cases in both domestic and wild species [6, 20–24]. As a result, only six cases of rabies were recorded in 2017 in the European Union (two cases each in Hungary, Romania and Poland) [25] and eight cases in 2018 (three cases in Romania, one in Lithuania and four in Poland) [26].

In the last few decades, Romania (238.397 km^2^) has been considered as one of European countries most affected by rabies [27, 28]. Since 2011, a national strategic programme [29] focusing on the surveillance and control of rabies in foxes has been implemented [30] through the biannual distribution of oral vaccine baits. In addition to oral vaccination campaigns, an important component of the rabies control programme in Romania is the mandatory vaccination of pets [31]. This strategy has led to a substantial decrease in the number of positive cases recorded in wildlife as well as in domestic animals [26–28].

As a comparison, in 2010 — before the fox vaccination programme was implemented — the whole country was infected, with a total number of 474 reported rabies cases, of which 339 were confirmed in wild animals and 135 in domestic animals [27]. In 2018 only three isolated rabies cases (one bovine, one dog and one fox) were registered [26].

Although the vaccine baits are intended for foxes, different non-target wild animals can also consume them [32–35]. Among wild animals involved in vaccine bait consumption, the most majority in Europe are wild boars [35]. A recent study in the USA [36] suggested a variety of non-target species other than feral swine and including coyotes and white-tailed deer as bait competitors. Naturally, animals infected with rabies virus do not survive, since the lethality reaches 100%, therefore the presence of rabies antibodies in wildlife is the result of vaccine baits consumption. The determination of rabies neutralising antibodies in the sera of animals sampled in vaccinated areas is a reliable indicator of the vaccination’s effectiveness [2, 37].

Given the few studies regarding the detection of rabies antibodies in wild boars and the absence of information concerning Romania, our study could also be considered as a complement to those already published. Furthermore, this study collected both fox and wild boar samples in vaccine-baited areas.

Worldwide, reference laboratories for rabies use serological tests to monitor and evaluate the efficacy and impact of ORV campaigns in the target species [2]. Considering a possible lack of consistency in the seroneutralisation tests on cells (i.e. the Fluorescent Antibody Virus Neutralisation Test (FAVN test) and Rapid Fluorescent Focus Inhibition Test-RFFIT) [2] for field sera collected from wild animals, this study used an ELISA since various factors such as cytotoxicity due to the use of cells, poor sample quality, environmental and carcass conditions prior to collection, or the time elapsed between the animal’s death and harvesting might influence the results with the FAVN test, an assay that uses live cell culture [35, 38, 39]. The BioPro Rabies ELISA Ab kit (Prague, Czech Republic), which was already validated for fox and raccoon dog sera, seemed to represent a valuable alternative to the FAVN test. The overall concordance between the two tests used on both species was high (95%), with the specificity for the ELISA reaching 100% [38]. This kit has also been tested on dog and cat sera in the framework of international trade and has proved to be highly specific. It also demonstrated an 86.2% concordance with the FAVN test [40]. However, this kit had never been previously used on wild boar serum or thoracic fluid samples.

In the last years, alternative techniques, represented by ELISA tests, have been developed and evaluated using field samples from red fox [38, 41–44] and raccoon dog [38]. In a 2010 review relating to the monitoring of ORV in the European Union, it was evaluated that 73% of the laboratories use an ELISA test, while 18% use RFFIT test and 9% FAVN test [45, 46]. As shown by Wasniewski et al. in 2013, samples collected from wild animals (in most cases body fluids) are taken in small quantities and may be cytotoxic for the cells [44, 47, 48]. In order to evaluate the efficacy of control measures, the serological data are not based on individual and accurate titres, but rather on global analysis in terms of seroprevalence, allowing estimation of whether or not herd immunity has been achieved at a certain time in a certain area [46]. The kit we evaluated in this study has also been tested recently on haemolytic thoracic fluid from foxes, with good results [39].

The objective of this study was to detect rabies antibodies in wild boar samples collected together with fox samples during the hunting season after ORV campaigns, in order to help determine whether wild boars are potential competitors for the vaccine baits.

## Results

### Detection of rabies antibodies by ELISA

#### In wild boar samples

All the tests were validated, with the positive and negative controls values found as expected, according to the criteria given by the manufacturer.

Out of the 312 wild boar samples tested, 180 (57.69%) were negative with a percentage of blocking (PB) below 40% (including 56 sera and 124 thoracic fluids) and 132 (42.31%) were positive, out of which 21 (6.73%) were positive with a PB between 43 and 70% and 111 (35.58%) were strongly positive with a PB ≥ 70% (ranging from 71 to 100%), equivalent to a value equal to or greater than 0.50 International Units per millilitre (IU/mL) (Table 1).

**Table 1:** Results of the wild boar and fox samples tested by ELISA (BioPro ELISA)

No. of tested samples / Species	PB < 40% (percentage negative)	% [95% CI]	PB 40–70% (percentage positive)	% [95% CI]	PB ≥ 70% (percentage strong positive)	% [95% CI]
312 wild boar samples	180 (57.69%)	57.7 [52.21–63.17]	21 (6.73%)	6.7 [3.95–9.51]	111 (35.58%)	35.6 [30.26–40.89]
345 fox samples	247 (71.60%)	71.6 [66.84–76.35]	46 (13.33%)	13.3 [9.75–16.92]	52 (15.07%)	15.1 [11.30–18.85]

*PB* Percentage of blocking.

*CI* Confidence Interval.

Among the 132 positive samples, 50 of them were represented by serum samples, while the other 82 were thoracic fluid samples. As concern the seropositivity per years, no positive sample was registered in 2014 (*n* = 10), in 2015 fourteen samples demonstrated rabies antibodies (14/68), while in 2016 a peak of seropositivity was recorded with 118 positive samples (118/234).

#### In fox samples

Out of the 345 fox samples tested, 247 (71.60%) were negative with a PB below 40% and 98 (28.40%) were positive, out of which 46 (13.33%) were positive with a PB between 40 and 70% and 52 samples (15.07%) were strongly positive with a PB ≥ 70% (ranging from 70 to 100%) (Table 1). Regarding the seropositivity per year in foxes, 26 samples (26/173) showed rabies antibodies in 2015, while 2016 was significantly higher with 72 positive samples out of 172.

### Detection of rabies antibodies by the FAVN test

#### In wild boar samples

##### Samples tested positive by ELISA

Of the 57 ELISA-positive samples (including 8 positive and 49 strongly-positive samples), 55 (96.49%) positive and two (3.51%) negative samples were obtained by the FAVN test (Table 2). The neutralising antibody titre of these two negative samples was equal to 0.02 IU/mL and to 0.22 IU/mL, respectively. A PB value of 88.44 and 83.91% was obtained respectively for the two samples with ELISA. A cytotoxic effect was identified on 11 samples during the FAVN test.

**Table 2:** Titre of rabies antibodies obtained by the FAVN test in wild boar samples

FAVN test					
Titre of antibody IU/ mL	< 0.5 IU/mL	0.5–1 IU/mL	1–2 IU/mL	2–5 IU/mL	≥ 5 IU/mL
No. of samples (%) (for the 57 samples tested positive by ELISA)	2 (3.51%)	6 (10.53%)	16 (28.07%)	20 (35.09%)	13 (22.80%)
No. of samples (%) (for the 44 samples tested negative by ELISA)	20 (45.45%)	10 (22.73%)	9 (20.46%)	5 (11.36%)	0 (0%)

##### Samples tested negative by ELISA

A total of 44 samples that tested negative by ELISA were tested by the FAVN test to check the ELISA kit’s specificity. Thus, 20 (45.45%) out of the 44 samples also gave a negative result with the FAVN test, while for the 24 (54.55%) remaining samples, the results were discordant with FAVN test results ranging from 0.5 to 4.6 IU/mL (Table 2). An additional file shows this in more detail (see Additional file 1).

It should be noted that for 20 samples, a cytotoxic effect was identified with the FAVN test due to the poor quality of the samples. Out of these 20 samples, 13 had a titre below 0.50 IU/mL (0.13–0.50 IU/mL), while for the other seven samples the titre varied between 0.66 and 1.99 IU/mL.

#### In fox samples

Fox samples were also tested by the FAVN test and compared to the results obtained with the wild boar samples in terms of percentage of immunisation.

##### Samples tested positive by ELISA

Of the 58 ELISA-positive samples (including 22 positive and 36 strongly-positive samples), 50 (86.21%) positive and eight (13.79%) negative samples were obtained by the FAVN test. A cytotoxic effect was identified on 22 samples during the FAVN test, but there was a correlation between the results from both tests for 21 out of 22 samples.

##### Samples tested negative by ELISA

As in the case of wild boars, most of the discordances between the two serological test results were observed on the ELISA-negative samples. Thus, of the 41 ELISA-negative samples, 15 (36.59%) negative and 26 (63.41%) positive samples were obtained with the FAVN test (Table 3). An additional file shows this in more detail (see Additional file 2). A cytotoxic effect was observed on 31 samples. For 10 out of these 31 samples, the results from both the FAVN test and ELISA were correlated, while for the remaining 21 samples, the results were different. Of the 26 samples that tested positive with the FAVN test, 25 of them had a titre between 0.50 and 4.56 IU/mL, while the remaining sample had a titre of 13.77 IU/mL.

**Table 3:** Detailed results of wild boar and fox samples tested in comparison by FAVN test and ELISA

Assay	Result	Wild boars	Foxes	Overall agreement with ELISA	
				Wild boars	Foxes
FAVN test (on ELISA-positive samples)					
	Positive ≥0.5 IU/mL	55	50	96.49%	86.21%
	Negative < 0.5 IU/mL	2	8		
	Total	57	58		
FAVN test (on ELISA-negative samples)					
	Positive ≥0.5 IU/mL	24	26	45.45%	36.59%
	Negative < 0.5 IU/mL	20	15		
	Total	44	41		

The ELISA results analysed in comparison to the FAVN test revealed a rate of false positives ranging from 3.51 to 13.79% and a rate of false negatives ranging from 54.55 to 63.41% for the wild boar and fox population, respectively.

#### Agreement between BioPro ELISA and the FAVN test

When 57 ELISA-positive and 44 ELISA-negative wild boar samples were also tested by the FAVN method, a significant statistical difference (*p* = 0.01) was noted, with an overall agreement between the two methods of 96.49% for positive and 45.45% for negative samples (Table 3).

The situation was similar for foxes, with 58 ELISA-positive and 41 ELISA-negative samples also tested by the FAVN method showing a significant statistical difference (*p* = 0.01), with an overall agreement of 86.21% for positive and 36.59% for negative samples (Table 3).

Concerning the ELISA-positive samples also analysed by the FAVN test, the overall agreement was higher in wild boars than in foxes, but not statistically significant (*p* = 0.094). The overall agreement between the two methods was 96.49% for wild boars and 86.21% for foxes (Table 3). Similarly, for the ELISA-negative samples from wild boars and foxes analysed by the FAVN test (*p* = 0.509), a large discrepancy was found. The overall agreement between the two methods for ELISA-negative samples was 45.45% for wild boars and 36.59% for foxes.

The overall concordance between the BioPro ELISA and FAVN test was 74.26% (75/101) for wild boar samples and 65.66% (65/99) for fox samples, where there was a correlation between the two methods for 140 out of 200 samples, although no significant statistical difference (*p* = 0.218) between the two species was registered. The same analysis conducted on samples not subject to cytotoxicity (data not shown) demonstrated an overall agreement between both tests of 72.86% for wild boar samples (51/70) and 73.91% for fox samples (34/46), with no statistical difference between the two species.

## Discussion

Mass vaccination programmes against rabies in wild animals are the only way to prevent, control and eliminate the disease in areas with a significant wildlife vector.

The oral vaccine baits used in Romania are live attenuated rabies virus with two dominant subpopulations, SAD (Street Alabama Dufferin) Bern and SAD B19 “like” (Lysvulpen Bioveta, Czech Republic) [30, 49].

Few studies aiming to detect rabies antibodies in wild boars have been carried out so far. The first study considering wild boars and roe deer was carried out in 1988 by Paquot et al. [32] in Belgium, where the vaccine’s biomarker (tetracycline is incorporated into the bait matrix to further evaluate the bait uptake in the target species) was found in the bones of these animals following the distribution of rabies vaccine baits (V-RG recombinant vaccinia-rabies virus), suggesting that these species are potential competitors for bait consumption.

Another study undertaken by Kierdorf and Ruhe in 2002 [34] found the vaccine’s biomarker in the teeth of two hunted wild boars. These two animals with tetracycline marks in the enamel of the permanent canines were found one in 1998 and the other in 2000, both in Germany. The presence of tetracycline was the result of the uptake of vaccine baits used for oral immunisation against rabies in one case and swine fever in the other. In Germany, three different SAD Bern-derived attenuated rabies virus vaccine strains — SAD B19, SAD P5/88 and SAD VA1 [50] — were used during this period for ORV campaigns.

A study undertaken [33] in France using two types of vaccine baits (Raboral V-RG and SAG1) showed that in addition to determining the tetracycline biomarker in the mandibles of wild boars, rabies antibodies were found in the sera of these animals in around 40.2% of the tested samples (43/107 samples). The RFFIT was used according to the method described by Smith et al., 1973 and modified by Zalan et al., 1979, with a positivity threshold of 0.4 IU/mL. Out of the 43 positive samples, 32.6% had a titre higher than 1 IU/mL.

The second study focusing on the detection of rabies antibodies in wild boars was performed by Vengust et al. in 2011 [35] in Slovenia. More samples were collected and rabies antibodies were detected in 28% (209/746) of the wild boar samples analysed. All 746 samples were tested through ELISA (Platelia, Bio-Rad, France), with a positivity threshold of 0.5 EU/mL and for a brief comparison 191/746 were also tested by the FAVN test, which found 122/191 (64%) to be positive. The rates are discordant, particularly when comparing data obtained with RFFIT [33] and the FAVN test [35]. Moreover, the study of Vengust et al. described a different ELISA kit (Platelia). In Slovenia, ORV campaigns have been implemented since 1988 with SAD Bern and SAD B19 vaccines [51]. It should be noted that studies conducted in Europe [19, 43, 52] performed on the Platelia Rabies II kit (Bio-Rad) reported a lower sensitivity of the test in animal samples. This could explain the low percentage found in Slovenia.

Both the FAVN test and the original RFFIT are considered reliable for evaluating the level of neutralising rabies antibodies [2]. However, in view of the disadvantages of these methods (which are costly, time-consuming and use live rabies virus so require suitably-equipped laboratories and well-trained personnel [40] in addition to the possible cytotoxic effect due to the use of cells [35]), they are more difficult to implement by laboratories involved in ORV monitoring using field samples.

Several ELISAs have proved to be a valuable method for detecting rabies antibodies in both the serum and thoracic fluid samples of hunted wild animals as alternatives to the serological tests using cell culture [35, 37–39]. BioPro’s ELISA was validated through an international study involving serum samples from foxes and raccoon dogs vaccinated with the different vaccines available in Europe [38, 53]. This test is currently being used in European countries where surveillance and control programmes for foxes are implemented, counteracting the effects of cytotoxicity and providing reliable results.

With respect to our results, when 312 wild boar samples of serum and thoracic fluid were tested by ELISA, 42.31% (132/312) were found to contain rabies antibodies. In order to check the diagnostic sensitivity and specificity of this ELISA on wild boar samples, ELISA-positive and -negative samples were also tested with the FAVN assay. For the ELISA-positive samples, 55/57 (96%) were positive by both methods, while a significantly higher discrepancy was observed between the two methods for the ELISA-negative samples, with only 45% of agreement. This is explained by cell cytotoxicity, which makes the interpretation of FAVN test results more difficult for negative sera (as the first wells of the microplate cannot be read) and consequently the titres obtained could be inconclusive [38]. It should be noted that the comparison of the PBs values with the IU/mL titres is not feasible, as there is no relation between the values obtained by both tests, because the level of positivity with the BioPro test is not linked to the rate of neutralising antibodies (meaning for example that a serum can have a PB value of 95% with a titre of 1 IU/mL and another serum can provide the same PB value with a titre of 20 IU/mL). Indeed, changing the cut off value of the ELISA would give a slight advantage on the specificity but not on the sensitivity; we did not choose this option, as the cut off has been previously demonstrated adequate for other wild animals – fox and raccoon dog [38].

Different factors could have influenced the results, such as the poor sample quality (blood or thoracic fluids may have been contaminated with other body fluids), environmental and carcass conditions prior to collection, but also the time elapsed from the animal’s death to harvesting (in a state of putrefaction or not), as has also been stated by other researchers [35, 39].

To summarise, 75/101 (74.26%) wild boar results were correlated when using both ELISA and FAVN methods. By removing the samples with cytotoxicity from the analysis (data not shown), the overall agreement was slightly decreased to 72.86% (51/70). Considering the limits of the FAVN test on very poor quality samples and also the objective of this study — to investigate a wild boar population for rabies antibody detection — this percentage of agreement was considered satisfactory.

Rabies antibody levels varied among samples, titres ranging from 0.13 IU/mL up to 31.55 IU/mL. This difference may be due to several situations: some wild boars may have ingested more baits (during the latest campaign or during previous campaigns), bit off only a small part of the bait or only punctured the vaccine blister. It may also reflect individual variations in immunological response [35].

In order to compare the wild boar results in terms of percentage of immunisation, fox samples (thoracic fluid) were also tested in the study.

All the fox samples were tested through ELISA and 28.40% of them revealed rabies antibodies following the ingestion of vaccine baits. The ELISA kit we used, already tested on fox thoracic fluid samples in Croatia [39], provided reliable results with a threshold of detection of 0.1 IU/mL. Even though the wild boar and fox samples were collected approximately in the same areas and the number of samples was similar (312 vs 345 respectively), the results obtained were unexpected. Although these baits were intended for foxes, wild boars exhibited a significantly higher percentage of positivity, with 132/312 (42.31%) samples showing rabies antibodies, compared to 98/345 for the fox results (28.40%). This low percentage of seropositive foxes is consistent with data published on the serological response of foxes sampled in Romania during the monitoring of ORV and tested by ELISA (Bio-Rad kit with a threshold of 0.5 EU/mL), with 29.29% seropositivity in 2016 [54]. The difference between the results obtained in wild boars and foxes cannot be explained by an effect due to sampling in the different locations. More studies are needed to be able to correctly interpret this observation taking into consideration the ecology of the wild boar population, and particularly animal density in vaccinated areas. The results found here on foxes tested with the BioPro ELISA clearly confirm the need to harmonise serological and sampling tools for assessing the immune response of wild animals collected in the field, as large discrepancies are observed between countries using similar tests [48, 53, 55].

For a brief comparison, some of the fox samples were also analysed by the FAVN test. An overall agreement of 65.66% (65 samples out of 99) was found between ELISA and FAVN methods. When ELISA-positive samples were tested by the FAVN test, more than 86% of results were correlated. When ELISA-negative samples were tested by the FAVN test, only 36.59% were also found negative. Cytotoxic effects due to the poor quality of the samples was also identified on fox samples, mostly in negative samples, which is the same situation observed in wild boars. This underlined the fact that the FAVN test is not suitable for poor-quality samples. The analysis conducted by removing cytotoxic samples (data not shown), allowed to improve the overall agreement on fox samples, obtaining a correlation between both tests of 73.91% (34/46).

Wild boars (*Sus scrofa*) are mammals living throughout Europe, Africa and most of Asia [56]. In Romania, the wild boar is one of the most widespread wild mammals, around 117.963 individuals being estimated across the country [57]. They are mainly found in forests from the Delta and Danube grasslands up to the Carpathians, but they can roam throughout the country [58]. Foxes and wild boars are generally found in the same areas, increasing the possibility for vaccine baits to be consumed by wild boars.

This is the first study showing that the BioPro ELISA might be used on wild boar samples and can provide satisfactory results compared to the FAVN test for this species. The results obtained by ELISA were not influenced by the quality of samples. The detection by ELISA of rabies antibodies in wild boar samples revealed a surprisingly high percentage of positive samples (42.31%). Our results are similar to those obtained in 1995 by Cliquet et al. [33], when 40.2% of the total samples contained rabies antibodies. To date, this study is the first to report the presence of rabies antibodies in wild boar samples collected in infected areas after ORV campaigns, during the hunting season in Romania.

Our study also highlighted the fact that wild boars represent an important factor in the implementation of oral fox vaccination, being without a doubt the main competitors (in line with the few studies conducted so far on this issue) for bait consumption. In areas where the density of these animals is very high, care should be taken to increase the number of baits in oral vaccination campaigns, when financially feasible [59] to ensure that enough foxes are vaccinated.

## Conclusions

Our own findings, along with those already in the literature, highlight the fact that wild boars represent a major risk for the proper execution of fox oral vaccination campaigns, being the main competitors for bait consumption.

Additional studies are needed to establish the influence of wild boars on implementing a successful plan for rabies elimination.

The BioPro ELISA kit has demonstrated to be a valuable tool to detect rabies antibodies in both serum and thoracic fluid of wild boar and fox samples. In infected countries, the need for harmonisation of sampling and serological tools could be undertaken by using this kit for assessing the immune response of wild animals collected in the field following ORV campaigns.

## Methods

### Ethical statement

The samples used in the study came from wild boars found dead or hunted within the national co-financed programme regarding the *“Monitoring, Control and Eradication of Classical Swine Fever”* [60] and from foxes hunted within the national *“Strategic Programme for Surveillance, Control and Eradication of Rabies in Foxes in Romania”* [29]. These documents constitute in Romania ethics permission as rabies is a notifiable disease and is engaged in European Commission funded eradication programmes for rabies and classical swine fever which include animal hunting for assessing their efficiencies.

As regards foxes, 45 days after the biannual distribution of vaccine baits, four foxes per year for each 100 km^2^ were hunted in each of the areas where these vaccination campaigns were held, in order to check campaign effectiveness. After these animals were hunted, their cadavers were packed up, stored cold with ice packs and sent directly to the authorised laboratory, Sanitary Veterinary and Food Safety Directorate (SVFSD) of Moldova Region, where brain samples were tested for rabies diagnosis through the direct fluorescent antibody (DFA) test [2]. If the results for rabies infection were negative, both mandibles (in order to be tested for tetracycline biomarker) and thoracic fluid were sampled in order to check vaccination effectiveness.

With respect to wild boar samples, organs and blood from the heart (if possible) or thoracic fluid were collected from those found dead in the field and sent to the laboratory for Classical Swine Fever testing (SVFSD of Moldova Region); the bodies were buried near the place where they were found. Wild boars that were hunted were transported to a wildlife collection centre and kept in appropriate temperature conditions until the laboratory results for Classical Swine Fever were available. In case these centres were not available, the samples were harvested immediately in the field, but in non-sterile conditions.

The hunting, sampling, packaging and transport of the samples to the laboratories were undertaken by authorised staff in compliance with national legislation and following the recommendations of international institutions [46, 61]. Across Europe, such procedures do not require any specific ethical approval, considering that hunting plans are part of the national disease control programmes. Depending on the availability of samples, the regional SVFSD of Iasi county in the region of Moldova, the SVFSD of Maramures, Buzau and Galati counties provided samples in order to conduct this study.

### Study area (58.580 km^2^)

**Fig. 1:**
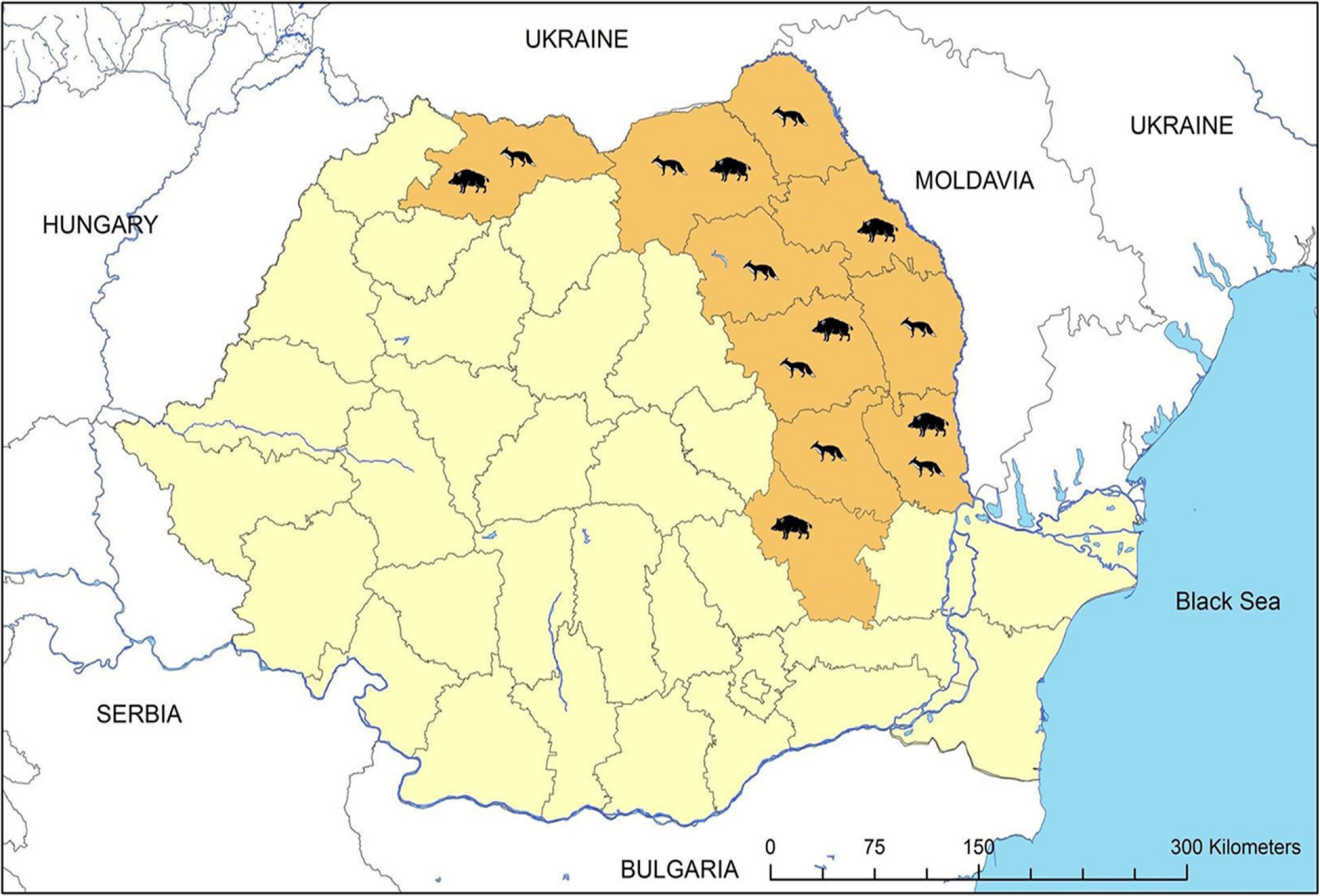
Map of Romania showing in orange the geographical origin of wild boar and fox samples. The location (in orange) of the wild boar and fox samples collected between 2014 and 2016 from the areas where oral rabies vaccination campaigns were undertaken. The map depicted in Fig. 1 is our own and was created using the ArcMap programme, version 10.5.1

The study was performed on samples collected from wild boars and foxes living in areas where ORV campaigns had been held. The areas consisted of counties located in north-eastern Romania, as shown in Fig. 1.

### Serum and thoracic fluid samples

The samples were collected between 2014 and 2016 (approximately at the same period in 2015 and 2016 for both species; in 2014 only wild boar samples (*n* = 10) were collected) in ten counties where ORV campaigns were undertaken, namely: Maramures, Suceava, Botosani, Iasi, Neamt, Vaslui, Bacau, Galati, Vrancea and Buzau, located in north-eastern Romania (Fig. 1).

A total of 312 wild boar samples collected in different animals (206 thoracic fluid and 106 serum samples) were tested. A total of 345 fox samples (thoracic fluid) were studied. Thoracic fluid was referring to the fluid found in the pleural cavity post-mortem.

Among the wild boar samples, 154 were from the county of Iasi, 106 from Galati, 27 from Buzau, 11 from Bacau, 10 from Maramures and four from Suceava.

Samples from foxes came from eight counties in the north-eastern part of Romania, namely: Neamt (*n* = 111), Bacau (*n* = 64), Vaslui (*n* = 49), Galati (*n* = 45), Botosani (*n* = 37), Vrancea (*n* = 22), Suceava (*n* = 11) and Maramures (*n* = 6).

The sera were extracted from blood samples by centrifugation and the thoracic fluid samples were stored at − 20 °C until use. All samples were heat inactivated, for 30 min at 56 ± 2 °C prior testing.

### Serological tests

All the samples were tested by ELISA. In order to assess the sensitivity and specificity of the ELISA serological test, 57 ELISA-positive and 44 ELISA-negative wild boar samples were also tested by the FAVN test, considered as the reference method [2, 62]. This assessment was also performed on fox samples (58 ELISA-positive and 41 ELISA-negative samples were also tested by the FAVN test). The fox and wild boar samples tested by both methods were those where a sufficient volume was available.

The FAVN test [62] consists of the neutralisation of a constant amount of RABV in vitro, using the challenge virus standard CVS strain, suitable for BHK-21 cell cultures, which are susceptible to the rabies virus [2].

ELISA is a rapid and simple serological test which consists in detecting rabies virus antibodies in the serum or plasma of domestic and wild carnivores and is a useful tool for monitoring rabies vaccination campaigns in wildlife species [2].

### Fluorescent antibody virus neutralisation test (FAVN test)

The method was performed according to the protocol described by [2, 62].

The DMEM growth medium was prepared and distributed in the wells of a 96-well microplate. Each control (CVS-11, cellular control, OIE positive reference serum of dog origin [63] and negative serum from a pool of unvaccinated dogs) as well as the samples to be tested were distributed in four consecutive wells and serially diluted. Fifty μL of challenge rabies virus (CVS – 11) containing around 100 TCID50 (50% Tissue Culture Infectious Dose) / 50 μL were added to each well.

The microplates were incubated for 1 h at 36 °C and then a volume of 50 μL of 4 × 10^5^ cells/mL of BHK-21 cell line suspension were distributed in each well and incubated at 36 °C for 48 h in a humidified incubator with 5% CO_2_.

After incubation, the microplates were visually checked for cytotoxicity. The content was discarded and the microplates washed with a sterile phosphate buffered saline – PBS. Acetone 80% was used for the first and second washes, then the microplates were left to rest for 30 min. After fixation, the content of the plates was discarded and air-dried under the biosafety cabinet. Each well was stained with 50 μL of an appropriate dilution of a fluorescein isothiocyanate (FITC) antirabies monoclonal globulin (Fujirebio Diagnostics, Malvern, USA), then the microplates were incubated for 30 min at 37 °C. The content was discarded, the microplates were washed with PBS, air-dried at room temperature for 10 min and read under the fluorescent microscope.

A qualitative reading was performed according to an “all or nothing” scoring method. The titre, expressed as International Units per millilitre (IU/mL) was calculated based on the Spearman-Kärber formula and a 0.50 IU/mL positivity threshold was used.

### Enzyme-linked immunosorbent assay (ELISA)

All samples (thoracic fluid and sera) were tested using a blocking ELISA (BioPro Rabies ELISA Ab Kit, BioPro, Prague, Czech Republic)*.* The titration was performed according to the manufacturer’s recommendations.

The method consisted in preparing the microplates coated with rabies antigen by bringing them up to room temperature before adding 50 μL of sample diluent to each well. The positive and negative controls, as well as the calibrated positive controls (CS1, CS2 and CS3, supplied by the manufacturer) were distributed in the wells in duplicate. Fifty microlitres of each sample was distributed in the wells and the plates were incubated overnight (18–24 h) at 2–8 °C with gentle shaking on an orbital shaker.

After overnight incubation, the content was discarded and the plates were washed six times with the washing solution before placing 100 μL of diluted biotinylated rabies antibody in each well. The plates were then incubated for 30 min at 37 °C with gentle shaking on an orbital shaker and then washed four times to remove the unbound biotinylated rabies antibodies. Next, 100 μL of diluted streptavidin peroxidase conjugate was added to each well and incubated for 30 min at 37 °C with gentle shaking and then washed four times to remove the unbound streptavidin peroxidase conjugate. After this, 100 μL of substrate solution (TMB) was added to each well forming a blue compound. The microplates were then incubated for 15–30 min at room temperature with gentle shaking, away from direct sunlight. The enzymatic reaction was stopped by adding 50 μL of stop solution (H_2_SO_4_). The optical density (OD) was read at 450 nm.

To validate the test, the OD for the negative control must be > 1 and the difference between means of OD of the negative and positive controls must be greater than or equal to 0.8. Values other than those recommended by the manufacturers would lead to invalidation of the results, hence the need to retest the samples.

The percentage of blocking (PB) should be between 45 and 70% for Control Serum 1 (CS1), between 25 and 45% for Control Serum 2 (CS2) and less than 30% for Control Serum 3 (CS3).

The PB for each sample was calculated according to the following formula:

$$ {\displaystyle \begin{array}{l} PB\%=O{D}_{NC}-O{D}_{sample}/O{D}_{NC}-O{D}_{PC}x\ 100\  where,\\ {}O{D}_{NC} represents\ the\ optical\ density\ of\ a\  negative\ control,\\ {}O{D}_{PC} represents\ the\ optical\ density\ of\ a\  positive\ control\ and\\ {}O{D}_{sample}, the\ optical\ density\ of\ the\ samples.\end{array}} $$

According to the manufacturer, a sample was considered positive when the PB was equal to or greater than 40% and negative when the PB was less than 40%. A PB equal to or greater than 70% was equivalent to 0.5 IU/mL corresponding to the FAVN test, the reference method. In order to evaluate the positivity of wild boar and fox samples, a PB value ≥40% was applied, this figure having already been used to evaluate the effectiveness of vaccination campaigns [38].

### Statistical analysis – agreement between ELISA and the FAVN test

Statistical analysis was performed using SPSS IBM version 21 software and Excel (version 2016) for the 95% Confidence Interval. Data was processed using the Shapiro-Wilk, Pearson Chi-Square and Fischer’s Exact tests. A “p” value of < 0.05 was considered statistically significant for all tests.

In order to evaluate the agreement between the two methods used in this study, 101 wild boar and 99 fox samples were selected and analysed by both methods. Agreement was defined as the ratio of positive and negative samples of both methods implemented, divided by the total number of samples tested.

## Supplementary information

**Additional file 1. **Wild boar samples (*n* = 101) tested by FAVN test and ELISA. Of the 57 ELISA-positive samples tested by the FAVN test, a cytotoxic effect was identified on 11 samples (marked with * in the table). Although cytotoxicity was seen, the results for the 11 samples were 100% correlated between FAVN test and ELISA. As concern the 44 ELISA-negative samples tested by the FAVN test, a cytotoxic effect was identified on 20 samples (marked with * in the table). For 13 out of these 20 samples, the results from both the FAVN test and ELISA were correlated, while for the remaining 7 samples, the results were different. (File format DOC Microsoft Word, size 21 KB)

**Additional file 2. **Fox samples (*n* = 99) tested by FAVN test and ELISA. Of the 58 ELISA-positive samples tested by the FAVN test, a cytotoxic effect was identified on 22 samples (marked with * in the table). For 21 out of these 22 samples, the results from both methods were correlated, while for the remaining sample, the result was different. As concern the 41 ELISA-negative samples tested by the FAVN test, a cytotoxic effect was identified on 31 samples (marked with * in the table). For 10 out of these 31 samples, the results from both the FAVN test and ELISA were correlated, while for the remaining 21 samples, the results were different. (File format DOC Microsoft Word, size 21 KB)

## Data Availability

The datasets used and/or analysed during the current study are available from the corresponding author on reasonable request.

## Abbreviations

BHK-21: Baby Hamster Kidney-21
CI: Confidence Interval
CO_2_: Carbon dioxide
CS1: Rabies control serum 1
CS2: Rabies control serum 2
CS3: Rabies control serum 3
CVS-11: Challenge Virus Standard-11
DMEM: Dulbecco’s Modified Eagle’s medium
ELISA: Enzyme-linked immunosorbent assay
EU: Equivalent Units
FAVN test: Fluorescent Antibody Virus Neutralisation test
FITC: Fluorescein isothiocyanate
H_2_SO_4_: Sulphuric acid
IU: International Unit
km^2^: Square kilometre
min: Minute
mL: Millilitre
nm: Nanometre
OD: Optical density
OIE: World Organisation for Animal Health
ORV: Oral rabies vaccination
PB: Percentage of blocking
PBS: Phosphate buffered saline
RABV: Rabies virus
RFFIT: Rabies Fluorescent Focus Inhibition Test
SAD strain: Street Alabama Dufferin strain
SVFSD: Sanitary Veterinary and Food Safety Directorate
TCID: Tissue Culture Infectious Dose
TMB: ELISA substrate solution
μL: Microlitre
°C: Celsius scale
