# Supplementary material for: Detection of rabies antibodies in wild boars in north-east Romania by a rabies ELISA test
Source: BMC Vet Res. 2019 Dec 21;15:466. doi: 10.1186/s12917-019-2209-x (PMC6925894; doi:10.1186/s12917-019-2209-x)
Supplement: Supplementary file 1 — Additional file 1. Wild boar samples (n = 101) tested by FAVN test and ELISA. Of the 57 ELISA-positive samples tested by the FAVN test, a cytotoxic effect was identified on 11 samples (marked with * in the table). Although cytotoxicity was seen, the results for the 11 samples were 100% correlated between FAVN test and ELISA. As concern the 44 ELISA-negative samples tested by the FAVN test, a cytotoxic effect was identified on 20 samples (marked with * in the table). For 13 out of these 20 samples, the results from both the FAVN test and ELISA were correlated, while for the remaining 7 samples, the results were different. (File format DOC Microsoft Word, size 21 KB) [file 12917_2019_2209_MOESM1_ESM.docx]

**Additional file 1:** **Wild boar samples (n=101) tested by FAVNt and ELISA.**

| **FAVNt vs ELISA (on 57 ELISA-positive samples)** | | | | **FAVNt vs ELISA (on 44 ELISA-negative samples)** | | | |
| --- | --- | --- | --- | --- | --- | --- | --- |
| No. of sample | Results ELISA  PB% | Results FAVN  UI/mL | Concordance | No. of sample | Results ELISA  PB% | Results FAVN  UI/mL | Concordance |
| 1 | 95.22 | 1.51 | Yes | 1* | 12.83 | 0.13 | Yes |
| 2 | 89.79 | 1.15 | Yes | 2 | 7.79 | 3.46 | No |
| 3 | 96.38 | 7.92 | Yes | 3* | 8.01 | 1.51 | No |
| 4 | 64.33 | 1.51 | Yes | 4 | 10.24 | 4.56 | No |
| 5* | 95.33 | 18.15 | Yes | 5* | 2.80 | 0.49 | Yes |
| 6 | 96.47 | 3.46 | Yes | 6* | 10.40 | 1.51 | No |
| 7 | 88.44 | 0.02 | No | 7* | 2.28 | 0.66 | No |
| 8 | 98.50 | 4.56 | Yes | 8* | 2.27 | 0.49 | Yes |
| 9 | 96.35 | 4.56 | Yes | 9* | 1.99 | 0.49 | Yes |
| 10 | 68.23 | 2.62 | Yes | 10* | 1.62 | 0.66 | No |
| 11 | 85.17 | 10.45 | Yes | 11* | 19.75 | 0.29 | Yes |
| 12 | 88.86 | 0.87 | Yes | 12 | 18.33 | 2.62 | No |
| 13 | 95.66 | 10.45 | Yes | 13* | 3.48 | 0.22 | Yes |
| 14 | 78.87 | 2.62 | Yes | 14* | 2.96 | 0.49 | Yes |
| 15 | 86.22 | 6.01 | Yes | 15 | 8.79 | 0.39 | Yes |
| 16 | 97.34 | 1.51 | Yes | 16 | 14.57 | 1.51 | No |
| 17 | 61.26 | 0.66 | Yes | 17 | 6.39 | 1.51 | No |
| 18 | 84.44 | 1.51 | Yes | 18* | 10.52 | 1.51 | No |
| 19 | 100.56 | 4.56 | Yes | 19* | 3.35 | 0.49 | Yes |
| 20 | 96.15 | 3.46 | Yes | 20 | 0.26 | 1.51 | No |
| 21 | 56.85 | 0.66 | Yes | 21* | 7.25 | 0.66 | No |
| 22 | 91.90 | 1.51 | Yes | 22* | 13.05 | 0.38 | Yes |
| 23 | 102.02 | 31.55 | Yes | 23 | 3.16 | 1.51 | No |
| 24 | 54.98 | 0.66 | Yes | 24 | 12.02 | 1.51 | No |
| 25 | 93.33 | 6.01 | Yes | 25 | 12.13 | 0.50 | No |
| 26 | 86.00 | 1.15 | Yes | 26* | 2.92 | 0.49 | Yes |
| 27 | 88.08 | 0.66 | Yes | 27 | 0.80 | 0.39 | Yes |
| 28 | 77.68 | 1.51 | Yes | 28 | 6.72 | 0.87 | No |
| 29 | 98.55 | 6.01 | Yes | 29 | 9.34 | 3.46 | No |
| 30* | 94.90 | 4.56 | Yes | 30 | 1.41 | 0.66 | No |
| 31 | 83.91 | 0.22 | No | 31 | 36.89 | 0.87 | No |
| 32 | 96.41 | 6.01 | Yes | 32* | 38.92 | 0.49 | Yes |
| 33 | 91.63 | 1.51 | Yes | 33* | 28.77 | 0.38 | Yes |
| 34 | 79.25 | 0.87 | Yes | 34* | 0.00 | 0.49 | Yes |
| 35* | 86.09 | 18.15 | Yes | 35 | 32.16 | 0.66 | No |
| 36 | 96.58 | 1.99 | Yes | 36 | 6.17 | 2.62 | No |
| 37 | 88.86 | 1.51 | Yes | 37 | 2.94 | 0.50 | No |
| 38* | 91.31 | 4.56 | Yes | 38 | 7.61 | 0.39 | Yes |
| 39* | 96.04 | 4.56 | Yes | 39 | 4.72 | 0.22 | Yes |
| 40 | 92.52 | 4.56 | Yes | 40 | 5.11 | 0.17 | Yes |
| 41 | 71.29 | 3.46 | Yes | 41 | 3.22 | 0.13 | Yes |
| 42* | 70.45 | 4.56 | Yes | 42 | 16.14 | 0.50 | No |
| 43 | 92.48 | 4.56 | Yes | 43 | 8.32 | 0.17 | Yes |
| 44 | 85.31 | 2.62 | Yes | 44* | 8.46 | 1.99 | No |
| 45 | 74.73 | 31.55 | Yes |  | | | |
| 46 | 98.93 | 13.77 | Yes |  |  |  |  |
| 47 | 96.89 | 13.77 | Yes |  |  |  |  |
| 48 | 95.22 | 4.56 | Yes |  |  |  |  |
| 49 | 99.55 | 3.46 | Yes |  |  |  |  |
| 50* | 49.92 | 1.51 | Yes |  |  |  |  |
| 51* | 100.08 | 1.51 | Yes |  |  |  |  |
| 52 | 47.07 | 1.51 | Yes |  |  |  |  |
| 53 | 87.80 | 2.62 | Yes |  |  |  |  |
| 54* | 86.87 | 1.15 | Yes |  |  |  |  |
| 55 | 96.21 | 2.62 | Yes |  |  |  |  |
| 56* | 75.30 | 2.62 | Yes |  |  |  |  |
| 57* | 44.64 | 1.15 | Yes |  | | | |

Legend

*: samples with cytotoxic effect on cell cultures by FAVNt.

Of the 57 ELISA-positive samples tested by the FAVNt, a cytotoxic effect was identified on 11 samples (marked with * in the table). Although cytotoxicity was seen, the results for the 11 samples were 100% correlated between FAVNt and ELISA.

As concern the 44 ELISA-negative samples tested by the FAVNt, a cytotoxic effect was identified on 20 samples (marked with * in the table). For 13 out of these 20 samples, the results from both the FAVNt and ELISA were correlated, while for the remaining 7 samples, the results were different.
