# Supplementary material for: Detection of rabies antibodies in wild boars in north-east Romania by a rabies ELISA test
Source: BMC Vet Res. 2019 Dec 21;15:466. doi: 10.1186/s12917-019-2209-x (PMC6925894; doi:10.1186/s12917-019-2209-x)
Supplement: Supplementary file 2 — Additional file 2. Fox samples (n = 99) tested by FAVN test and ELISA. Of the 58 ELISA-positive samples tested by the FAVN test, a cytotoxic effect was identified on 22 samples (marked with * in the table). For 21 out of these 22 samples, the results from both methods were correlated, while for the remaining sample, the result was different. As concern the 41 ELISA-negative samples tested by the FAVN test, a cytotoxic effect was identified on 31 samples (marked with * in the table). For 10 out of these 31 samples, the results from both the FAVN test and ELISA were correlated, while for the remaining 21 samples, the results were different. (File format DOC Microsoft Word, size 21 KB) [file 12917_2019_2209_MOESM2_ESM.docx]

**Additional file 2: Fox samples (n=99) tested by FAVNt and ELISA.**

| **FAVNt vs ELISA (on 58 ELISA-positive samples)** | | | | **FAVNt vs ELISA (on 41 ELISA-negative samples)** | | | |
| --- | --- | --- | --- | --- | --- | --- | --- |
| No sample | Results ELISA  PB% | Results FAVN  UI/ml | Concordance | No sample | Results ELISA  PB% | Results FAVN  UI/ml | Concordance |
| 1* | 81.06 | 1.51 | Yes | 1* | 1.59 | 0.50 | No |
| 2 | 81.61 | 1.99 | Yes | 2 | 0.78 | 13.77 | No |
| 3 | 59.59 | 0.50 | Yes | 3* | 19.08 | 4.56 | No |
| 4 | 81.57 | 1.99 | Yes | 4* | 6.27 | 4.56 | No |
| 5* | 90.73 | 1.99 | Yes | 5* | 4.80 | 1.51 | No |
| 6* | 94.74 | 4.56 | Yes | 6* | 15.58 | 0.87 | No |
| 7 | 75.42 | 3.46 | Yes | 7* | 13.09 | 4.56 | No |
| 8 | 90.86 | 1.51 | Yes | 8* | 18.24 | 0.87 | No |
| 9 | 80.28 | 0.17 | No | 9* | 20.88 | 0.87 | No |
| 10 | 83.12 | 0.22 | No | 10* | 25.10 | 0.22 | Yes |
| 11 | 40.65 | 0.50 | Yes | 11 | 5.32 | 1.51 | No |
| 12 | 81.85 | 1.51 | Yes | 12* | 7.89 | 1.51 | No |
| 13 | 42.42 | 0.17 | No | 13* | 0.89 | 0.66 | No |
| 14 | 55.54 | 0.06 | No | 14* | 16.77 | 1.15 | No |
| 15* | 83.75 | 2.62 | Yes | 15* | 10 | 0.66 | No |
| 16 | 96.06 | 1.51 | Yes | 16* | 3.21 | 0.49 | Yes |
| 17 | 90.53 | 0.29 | No | 17* | 5.39 | 0.49 | Yes |
| 18* | 89.35 | 1.51 | Yes | 18 | 3.21 | 0.49 | Yes |
| 19 | 49.56 | 2.62 | Yes | 19* | 2.12 | 0.17 | Yes |
| 20* | 92.67 | 13.77 | Yes | 20* | 3.24 | 0.17 | Yes |
| 21 | 56.98 | 1.15 | Yes | 21 | 2.56 | 1.51 | No |
| 22 | 95.41 | 1.99 | Yes | 22* | 8.93 | 0.29 | Yes |
| 23 | 82.96 | 4.56 | Yes | 23* | 0.44 | 0.49 | Yes |
| 24* | 70.44 | 1.99 | Yes | 24* | 10.70 | 1.51 | No |
| 25 | 75.09 | 0.50 | Yes | 25* | 12.47 | 1.51 | No |
| 26 | 89.13 | 4.56 | Yes | 26* | 12.58 | 0.22 | Yes |
| 27* | 61.47 | 0.50 | Yes | 27* | 3.52 | 1.51 | No |
| 28* | 65.81 | 1.51 | Yes | 28* | 36.22 | 4.56 | No |
| 29* | 89.01 | 31.55 | Yes | 29* | 0.30 | 0.87 | No |
| 30 | 95.00 | 0.39 | No | 30* | 7.62 | 1.51 | No |
| 31 | 45.13 | 1.51 | Yes | 31 | 12.31 | 0.06 | Yes |
| 32 | 92.35 | 1.51 | Yes | 32* | 5.02 | 1.51 | No |
| 33 | 41.43 | 1.51 | Yes | 33 | 1.59 | 0.06 | Yes |
| 34 | 82.87 | 2.62 | Yes | 34* | 36.63 | 1.51 | No |
| 35 | 50.47 | 1.15 | Yes | 35* | 32.25 | 0.66 | No |
| 36 | 68.82 | 1.99 | Yes | 36 | 15.80 | 0.29 | Yes |
| 37 | 69.15 | 1.51 | Yes | 37* | 1.10 | 0.39 | Yes |
| 38 | 59.14 | 1.15 | Yes | 38 | 3.16 | 1.51 | No |
| 39 | 81.68 | 4.56 | Yes | 39 | 15.50 | 0.50 | No |
| 40 | 83.17 | 1.51 | Yes | 40* | 0.39 | 0.17 | Yes |
| 41 | 89.00 | 3.46 | Yes | 41 | 8.50 | 0.06 | Yes |
| 42 | 74.48 | 4.56 | Yes |  |  |  |  |
| 43* | 54.52 | 4.56 | Yes |  |  |  |  |
| 44 | 68.46 | 1.51 | Yes |  |  |  |  |
| 45* | 65.86 | 0.50 | Yes |  |  |  |  |
| 46* | 71.94 | 10.45 | Yes |  |  |  |  |
| 47* | 70.39 | 4.56 | Yes |  |  |  |  |
| 48* | 55.56 | 1.51 | Yes |  |  |  |  |
| 49 | 81.06 | 0.50 | Yes |  |  |  |  |
| 50 | 71.10 | 0.50 | Yes |  |  |  |  |
| 51* | 59.60 | 4.56 | Yes |  |  |  |  |
| 52* | 57.14 | 4.56 | Yes |  |  |  |  |
| 53* | 60.26 | 1.51 | Yes |  |  |  |  |
| 54* | 90.49 | 4.56 | Yes |  |  |  |  |
| 55* | 88.97 | 0.66 | Yes |  |  |  |  |
| 56* | 86.42 | ≤0.50 | Yes |  |  |  |  |
| 57 | 49.98 | 0.06 | No |  |  |  |  |
| 58* | 123.79 | 0.29 | No |  |  |  |  |

Legend

*: samples with cytotoxic effect on cell cultures by FAVNt.

Of the 58 ELISA-positive samples tested by the FAVNt, a cytotoxic effect was identified on 22 samples (marked with * in the table). For 21 out of these 22 samples, the results from both methods were correlated, while for the remaining sample, the result was different.

As concern the 41 ELISA-negative samples tested by the FAVNt, a cytotoxic effect was identified on 31 samples (marked with * in the table). For 10 out of these 31 samples, the results from both the FAVNt and ELISA were correlated, while for the remaining 21 samples, the results were different.
